# Artificial Caries Resistance in Enamel after Topical Fluoride Treatment and 445 nm Laser Irradiation

**DOI:** 10.1155/2019/9101642

**Published:** 2019-11-03

**Authors:** Mohammed Abbood Al-Maliky, Matthias Frentzen, Jörg Meister

**Affiliations:** ^1^Department of Periodontology, Operative and Preventive Dentistry, Dental Faculty, University of Bonn, Bonn, Germany; ^2^Center of Applied Medical Laser Research and Biomedical Optics (AMLaReBO), Bonn University, Bonn, Germany; ^3^Department of Biomedical Applications, Institute of Laser for Postgraduate Studies, University of Baghdad, Baghdad, Iraq

## Abstract

**Objective:**

This in vitro study is aimed at investigating the caries preventive effectiveness of 445 nm diode laser in combination with topical fluoridation.

**Materials and methods:**

A total of 30 caries-free bovine teeth were used in this study. Eighteen teeth were covered with nail varnish except four windows on the labial surface. The windows were assigned to no treatment/control (C), laser (L) (0.3 W, 60 s, and 90 J/cm^2^), fluoride (F), and fluoride followed by laser (FL) treatment groups. Artificial caries lesions were created, and the teeth were sectioned and investigated under polarized light microscopy for quantitative measurement of the resulted lesion depth. Ten teeth were used for surface temperature measurement and two teeth for scanning electron microscopy (SEM). Extra twelve human molars were used for the intrapulpal temperature measurement. The absorbance of fluoride at 445 nm was measured.

**Results:**

The means of lesion depth for the C, L, F, and FL groups were 123.48 (±21.93), 112.33 (±20.42), 99.58 (±30.68), and 89.03 (±30.38) *μ*m, respectively. The pairwise differences of the L, F, and FL groups compared with the C group were significant (*p* < 0.05). The differences between groups were tested: FL versus L *p*=0.02, F versus L *p*=0.16, and FL versus F *p*=0.91, and the difference of the F versus FL was not significant (*p*=0.91). Temperature increment at the enamel surface and pulp roof were ∆*T* = 16.67 (±4.11) and 2.12 (±0.66)°C, respectively. The topical fluoride absorbance at 445 nm is five orders higher than that at 810 nm. SEM shows that after laser irradiation the enamel surface was intact and without thermal damage.

**Conclusions:**

The 445 nm laser irradiation may be useful for caries prevention, and its effectiveness is lower than those previously achieved using the argon ion laser.

## 1. Introduction

Dental caries is the most widespread noncommunicable disease globally, affecting half of world's population and caused by demineralization of dental tissues by acids produced as a byproduct from the cariogenic bacteria during carbohydrates fermentation. Everybody is at risk of dental caries, and children and adolescents are the most affected categories, and it consumes about 5–10% of the healthcare financial plan in the industrialized countries [1].

The inorganic components of enamel are composed of (86%), water (12%), and organic components (< 2%), and the organic components are composed mainly of proteins (Amelogenin and enamelin) and lipids surfacing the enamel prisms together with the water [2]. The inorganic component is carbonated hydroxyapatite (Ca_10_(PO_4_)_6−*X*_(OH)_2−*Y*_)(CO_3_)_*X*+*Y*_. Similar to other mineralized biological tissues, hydroxyapatite has many variations when compared with the stoichiometric hydroxyapatite (Ca_10_(PO_4_)_6_(OH)_2_), and these variations include missing ions like calcium and about 20–30% of hydroxyl; fluoride ion fills the hydroxyl vacancies or replaces existing ones and reduces the solubility product of the enamel [3].

Fluoride is the most effective method for caries prevention which can be used either topically in the form of gels, vanishes, fluids, and tooth pastes or systemically like in water fluoridation and table salts; besides, fluoridated sealants are used for covering occlusal pits and fissures. Furthermore, lasers were used in research for caries prevention since 1965 by Sognnaes and Stern [4], and they were employed in different treatment approaches like laser irradiation alone or in combination with fluoride. Lasers used for caries prevention in combination with fluoride include argon ion [5], CO_2_ [6], Er:YAG [7], Er, Cr:YSGG [8], Nd:YAG [6], and diode [9] lasers, and their results were promising due to their ability to alter the enamel chemically, or morphologically in the case of using higher doses, and this alteration renders the enamel to be more acid resistant. However, the results of the previous studies are variable depending on the type of laser system, study design, and investigation method. Argon (488 nm or 488 + 514 nm) laser was used since two decades for this purpose with encouraging results ranging from 15–46% of caries reduction with laser alone and 25–55% for the combined laser-fluoride treatment [10–12]. This study is attempted at testing a hypothesis that the 445 nm laser could be a close wavelength to that of the argon ion laser and may have a caries preventive effect when combined with fluoride [10, 12].

The biophysical properties of the 445 nm wavelength compared with the conventional diode lasers in the range of 810–980 nm include a higher absorption by pigmented tissues (*μ*_a_ > 1000 cm^−1^ compared with ∼10 cm^−1^) and collagen but slightly different absorption properties in dental hard tissue. Additionally, the absorption of this laser by water (*μ*_a_ < 0.001 cm^−1^) is lower than those of the infrared diode lasers (*μ*_a_ > 0.01 cm^−1^). Furthermore, scattering increases tremendously between 700–220 nm from 20 to 400 cm^−1^ [13]. It has a high efficiency for soft tissue cutting and also for root canal disinfection. Also, it has been suggested that proteins and enzymes containing riboflavin and porphyrins act as photoreceptors for blue-violet wavelengths [14, 15]. Blue laser or LED light sources have shown biomodulatory properties like wound healing and expression of chodrogenic mRNA by prechondrogenic cells [16, 17]. For lasers that have high absorption coefficients (*μ*_a_ > 1000 cm^−1^) in hydroxyapatite like CO_2_ and Er : YAG lasers, the absorption will be restricted to the first few micrometers of the surface of enamel where temperature can reach 1000°C. In the lasers of the visible spectrum, where the absorption coefficient in hydroxyapaptite is weak (*μ*_a_ < 1 cm^−1^), the interaction with enamel is mainly dependent on scattering rather than on absorption and a much lower surface temperature is expected. To understand the laser-enamel interaction of the 445 nm diode laser, the 810 nm was used as a representative for the infrared diode lasers for comparison.

The aim of this in vitro study is the investigation of a not yet been tested 445 nm diode laser for caries prevention alone and in combination with topical fluoridation.

## 2. Materials and Methods

### 2.1. Sample Preparation and Grouping

**Figure 1:**
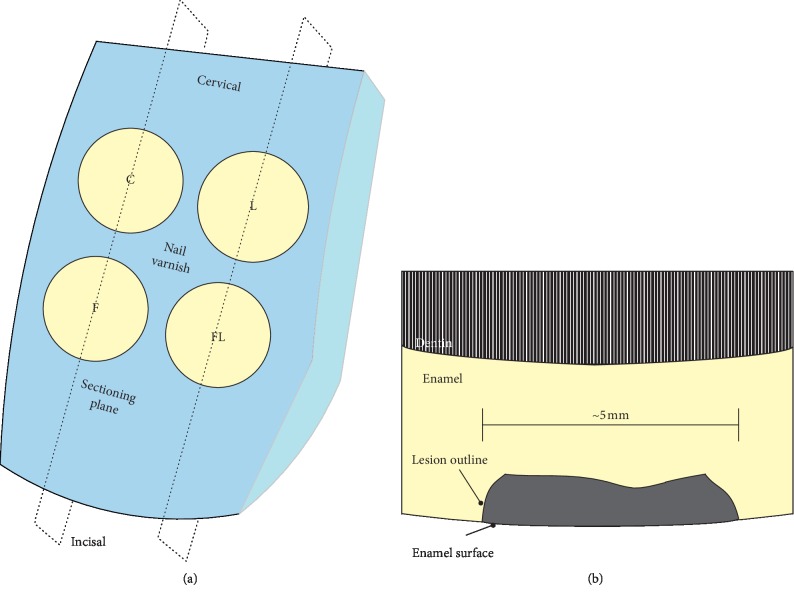
(a) Illustration of the treatment windows and sectioning planes. No treatment/control (C), laser (L), fluoride (F), and fluoride followed by laser (FL). (b) Illustration of the tooth section as shown under the polarized light microscopy including the lesion and the anatomical landmarks.

Eighteen caries-free bovine teeth, stored in 0.9% NaCl with 0.001% sodium azide at 4°C, were polished by nonfluoridated pumice (Cleanic™ RDA 27, Kerr GmbH, Biberach, Germany) for 10 s and covered with nail varnish except four windows (5 mm in diameter) on the labial surface. The windows were assigned to no treatment/control (C), laser (L), fluoride (F), and fluoride followed by laser (FL) treatment groups. Windows were allocated in a turnover experimental design. In this design, each treatment group has equal number of windows located mesially, distally, gingivally, and incisally to avoid possible influence from window position (Figure 1(a)).

### 2.2. Fluoride Treatment

The F and FL groups were treated with topical fluoride elmex®*fluid* (CP GABA GmbH, Germany) containing 1% aminfluoride (10,000 ppm and pH 3.9) for 3 min. Then, each treatment window was rinsed by 2.5 ml distilled water to remove excess fluoride and dried carefully with tissue paper. Each sample was treated carefully and individually to avoid contamination of the nonfluoridated windows. In addition, the surface tension of the fluoride liquid restricted the fluoride to the borders of nail varnish. At this point, the FL group was treated by laser.

### 2.3. Laser Treatment

The L and FL groups were irradiated with a prototype laser system that contains two modules of 445 nm and 810 nm diode lasers in the same machine (A.R.C. Laser, Nuremberg, Germany). Low-level irradiation settings were used: 0.3 W, 60 s, and 5 mm spot size and 320 *μ*m fiber diameter and 90 J/cm^2^ dose in CW irradiation mode (Table 1). The laser handpiece was fixed by a universal laboratory clamp so that the fiber is perpendicular to the enamel surface, and the teeth were fixed by heavy body silicon to *xyz* table for accurate positioning. An isolating art paper with 5 mm window was used to restrict laser irradiation to the treatment windows. The output power and the transmission of the isolating paper were verified by a power meter (LabMax Top, Coherent Inc., Santa Clara, CA, USA). After irradiation, the teeth were stored in normal saline during the weekend to be rehydrated before the immersion in the pH cycling solutions.

### 2.4. pH-Cycling Procedure

A simulated caries process consisting of 5 days of pH-cycling was implemented; each day includes 6-hour demineralization followed by 18-hour remineralization. Afterward, teeth were immersed in the remineralization solution for 2 days [18]. The demineralization solution contains 2.0 mmol l^−1^ Ca, 2.0 mmol l^−1^ P, 0.075 mol l^−1^ acetate buffer, and 0.04 ppm F at pH 4.7. The remineralization solution contains 1.5 mmol l^−1^ Ca, 0.9 mmol l^−1^ P, 0.15 mol l^−1^ KCl, 0.02 mol l^−1^ cacodylate buffer, and 0.05 ppm F at pH 7.0. The proportion of the demineralization and remineralization solutions to the area of enamel surface was 2.22 and 1.11 ml/mm^2^, respectively [18]. The whole procedure was performed at 37°C, and the samples were immersed individually in the mentioned solutions. The samples were washed with distilled water for 10 s and gently dried with tissue paper when replaced from the demineralization to the remineralization solution and vice versa.

### 2.5. Polarized Light Microscopy

The teeth were sectioned using a precision sectioning machine (EXAKT 300 CPV, Norderstedt, Germany) to obtain ∼400 *μ*m thick slices. The resulted sections were manually polished with #500 and #1200 carbide-paper to a thickness of 250 (±24) *μ*m. Each section was imbibed in distilled water and mounted on glass slides and covered with cover slips to be ready for microscope examination (Dialux 20 EB, Leica Microsystems, Wetzlar, Germany). Two polarization filters were mounted in both sides of the section with a *λ* filter placed over the light source with a standardized samples position. The microscope was used in the polarization mode and with maximum illumination at a magnification of ×16. Then, photomicrographs were recorded using an attached camera (Leica DFC420C, Leica Microsystems, Wetzlar, Germany). Lesion depth measurements conducted using computer software (ImageJ 1.51K, NIH, USA), and the averaged lesion depth of each window was determined by measuring a lesion area of 4000 *μ*m in length then divided by 4000 [19] (Figure 1(b)).

### 2.6. Surface Temperature Measurement

Ten bovine teeth were used for measuring the surface temperature. A thermal camera was used for this purpose (VarioCAM® hr, InfraTec. GmbH, Dresden, Germany) using the near-field lens of 50 mm focal length. The experiment was conducted at room temperature of 22°C. A data acquisition program was used for results recording (IRBIS® V2.3, InfraTec. GmbH, Dresden, Germany) and saved as Excel files. The recorded results of the 445 nm diode laser was compared with those obtained during lasing with 810 nm diode laser (A.R.C. Laser, Nuremberg, Germany) using the same teeth and irradiation parameters. The 810 nm diode laser was selected as a representative of the infrared (IR) of diode lasers. This comparison was done to evaluate the laser-enamel interaction of the 445 nm laser.

### 2.7. Pulp Temperature Measurement

**Figure 2:**
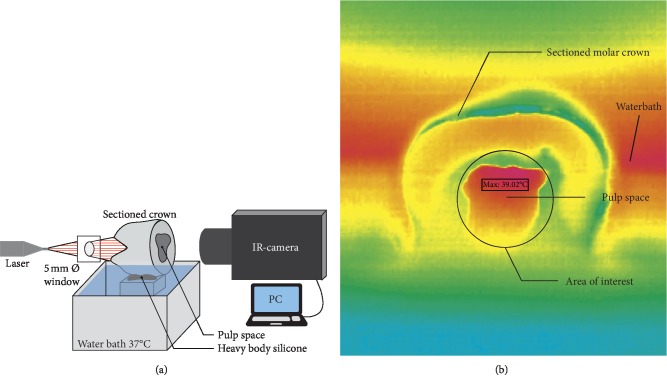
(a) Illustration of the temperature measurement test. The teeth crowns are mounted on a table containing heavy body silicone material and part of the crown is immersed in the water to allow heat conduction. (b) Infrared thermal image. An example thermal image for the maximum intrapulpal temperature recording for one of the samples. The circle represents the selected area of interest for recording the temperature at the pulp roof.

Twelve human molars were used for testing the intrapulpal temperature increment. After sectioning at ∼1 mm gingival to the cementoenamel junction, teeth crowns were mounted on a table containing heavy body silicone material as stabilizer for accurate crowns positioning inside a water bath (MWB, JULABO GmbH, Seelbach, Germany). The crowns were positioned in a way that part of the crown was immersed in water to be heated to 37 (±0.23)°C. The occlusal surface of the crowns was facing the laser fiber, and the pulpal side was facing the thermal camera (VarioCAM® hr, InfraTec. GmbH, Dresden, Germany) (Figure 2). The same laser irradiation settings described in Table 1 were employed in this test, and the 5 mm spot size was realized by a window of an art paper barrier to avoid irradiation of the adjacent enamel. The temperature recording was started at 5 s before irradiation and continued for 25 s after the irradiation end. A near-field lens of 50 mm focal lens was mounted on the camera for this purpose. Data acquisition was set at 25 Hz, and data storage was done by a software package (IRBIS® V2.3, InfraTec. GmbH, Dresden, Germany) as Excel sheet files. The maximum reading for each sample was used for further statistical analysis. The crown thickness, starting from the roof of the pulp to the central fossa of the occlusal surface, was measured using an incremental thickness gauge (ORBIS Dental Handelsgesellschaft mbH, Münster, Germany).

### 2.8. Spectral Absorption of the Topical Fluoride

The light absorbance of the topical fluoride agent used in this experiment was measured with a spectrophotometer (Epoch, BioTek instruments GmbH, Bad Friedrichshall, Germany). The spectrophotometer was calibrated at 445 nm and 810 nm wavelengths, and the measurements (*n* = 6) were done for both wavelengths. The absorbance of distilled water (Aqua, B. Braun Melsungen AG, Melsungen, Germany) was also measured at these wavelengths to be used as a reference to the topical fluoride results. The absorbance was measured by the spectroscopic machine by measuring light loss through a 3 mm thick layer of the topical fluoride agent.

### 2.9. SEM Test

The bovine teeth were prepared, fluoridated, and irradiated with the same methodology described in Sections 2.1to 3 (*n* = 2). The treated teeth were fixed with 4% paraformaldehyde in phosphate-buffered saline. The teeth dehydration was done by using increasing ethanol-alcohol concentrations. These concentrations were started at 30, 50, and 70% 2x for 1 h then 80% overnight and followed by 90, 95, and 100% for 24 h each. After being dried for 24 h, the teeth were platinum sputtered to be investigated with SEM (XL 30, Philips, Eindhoeven, Netherlands) at 25 kV.

### 2.10. Statistical Analysis

IBM SPSS 21.0 (IBM Corp., Armonk, NY, USA) was used for the statistical analysis and for the descriptive parameters calculation. Regarding the polarized light microscopy (PLM), the data were tested by Shapiro–Wilk's test for normality and by Mauchly's test for sphericity. For the assessment of the potential preventive effects of the experiment groups, a one-way repeated measures analysis of variance (RM-ANOVA) with Bonferroni adjustment was used. The G^*∗*^Power V. 9.1.9.2 program was used for the sample size selection with an effect size *f* of 0.4. For the surface temperature data, a paired samples *t* test with Bonferroni correction was applied. While for the pulpal temperature measurement, one-sample *t* test was used to test whether the obtained results were significantly lower than the classical pulp damage threshold of 5.6°C [20]. The 5.6°C temperature increment was used as a theoretical expectation for the null hypothesis. The correlation between crown thickness and the recorded pulp temperature increment was verified by the Pearson correlation test. The significance level was set at *p*=0.05.

## 3. Results

### 3.1. Lesion Depth Measurement

**Figure 3:**
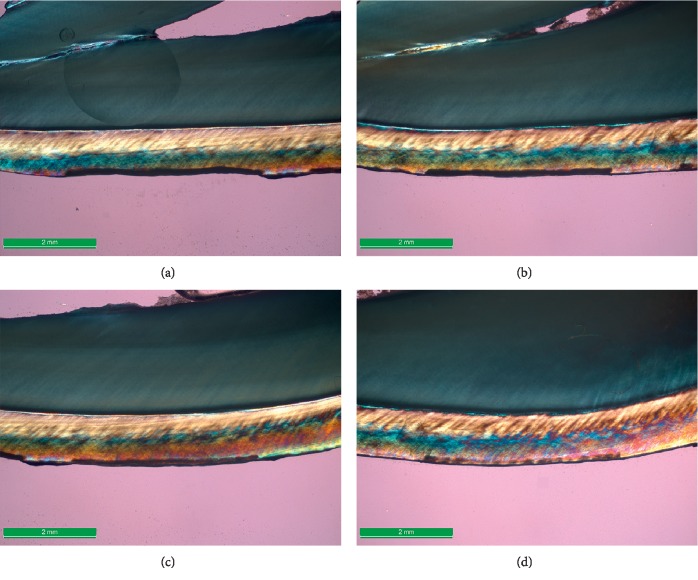
Polarized light microscopy of the paired representative lesion (water imbibition, ×16). (a) No treatment/control (C), (b) 445 nm laser irradiation alone, (c) topical fluoridation alone, and (d) topical fluoridation followed by 445 nm laser irradiation.

**Figure 4:**
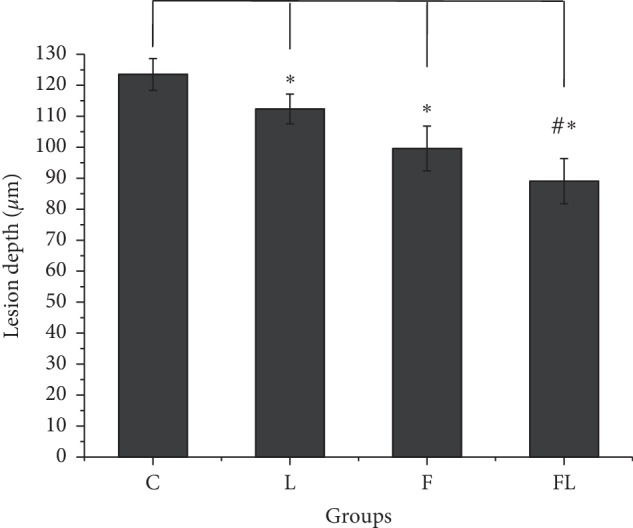
Means of lesion depth of the polarized microscopy experiment (±SE). No treatment/control (C), laser (L), fluoride (F), and fluoride followed by laser (FL) treatment groups. The symbol “∗” represents a statistical difference when compared with the C group at *p* < 0.05. The symbol “#” represents a statistical difference when compared with the L group at *p* < 0.01, *n* = 18.

The mean and standard deviation of the lesion depth measured by PLM for the C, L, F, and FL groups were 123.48 (±21.93), 112.33 (±20.42), 99.58 (±30.68), and 89.03 (±30.38) *μ*m, respectively (Figures 3 and 4) (Table 2). The Shapiro–Wilk test showed a normal distribution of the group's residuals, and Mauchly's test of sphericity showed a *p* value of 0.11. The results of the RM-ANOVA indicated a significant preventive effect, *F*_(3,51)_ = 12, 58, *p* < 0.01. Follow-up pairwise comparisons indicated that the difference of the L, F, and FL groups compared with the C group was significant, *p* < 0.05. The difference between the FL and L groups was significant, *p* < 0.01, while the difference between the F and L was not significant *p*=0.16. Finally, there was no significance difference between the FL and F groups, *p*=0.91. The laser treatment alone provided a preventive effect of 9% against enamel demineralization, whereas the combination of fluoride and laser provided 27.9%, which was higher than the 19.4% achieved by the fluoride treatment alone.

### 3.2. Surface Temperature Analysis

**Figure 5:**
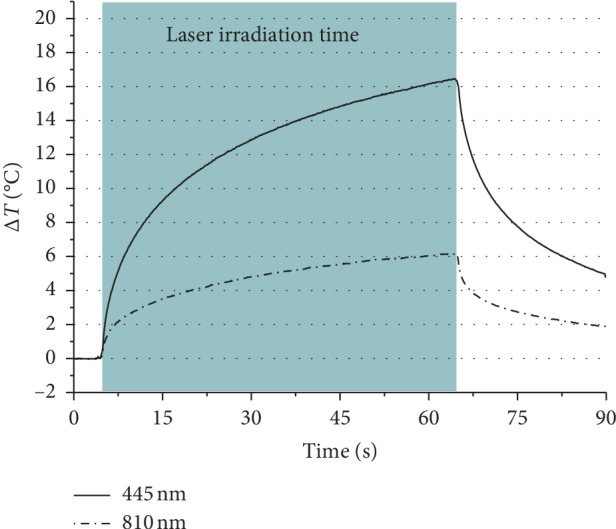
Mean value of all tested teeth in paired real time surface temperature increment of enamel during the 445 and 810 nm diode lasers irradiation (0.3 W 60 s and a dose of 90 J/cm^2^).

The paired samples *t* test showed a significant difference between the 445 nm and 810 nm lasers, *p* < 0.01. The mean and standard deviation of the enamel ∆T surface temperature increment of the 445 nm and 810 nm lasers were 16.67 (±4.11) and 6.26 (±0.54)°C, respectively. The 445 nm laser irradiation of enamel led to 62.45% higher surface temperature increase than that of the 810 nm laser (Figure 5) (Table 3).

### 3.3. Pulpal Temperature Analysis

**Figure 6:**
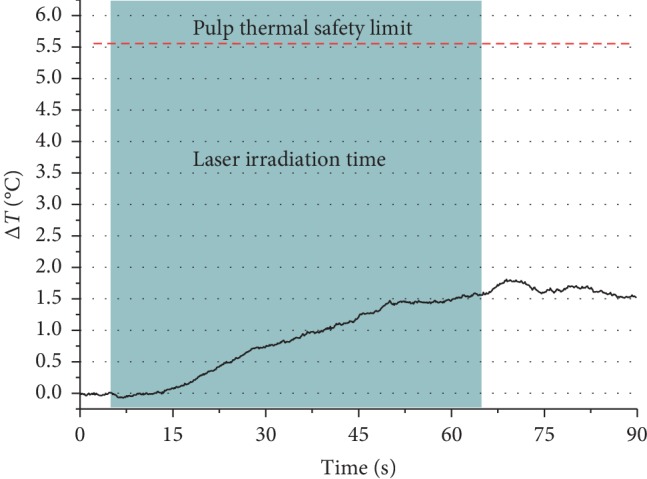
Mean real time temperature increment in the roof of the pulp chamber during the 445 nm laser irradiation (0.3 W 60 s and a dose of 90 J/cm^2^). The shaded area represents the time of laser irradiation on the occlusal surface. The peak temperature shown in this graph is lower than the mean of the maximums of 2.12°C; this is due to that the samples reach their maximum intrapulpal temperature at different times leading to a lower-averaged curve.

The mean of the temperature increment measured at the roof of the pulp chamber was 2.12°C (±0.66), and the maximum recorded temperature was 2.97°C. The recorded temperature was significantly lower than the thermal damage threshold of the pulp (5.6°C), t_(11)_ = 18.266, *p* < 0.01. The corresponding mean of the crowns thickness starting from the roof of the pulp to the central fossa of the occlusal surface was 4.10 mm (±0.38) (Table 4) (Figure 6). The Pearson correlation test showed a negative relation between the crown thickness and the recorded temperature increment (*R* = −0.648, *p*=0.023).

### 3.4. Spectral Absorbance Analysis

**Figure 7:**
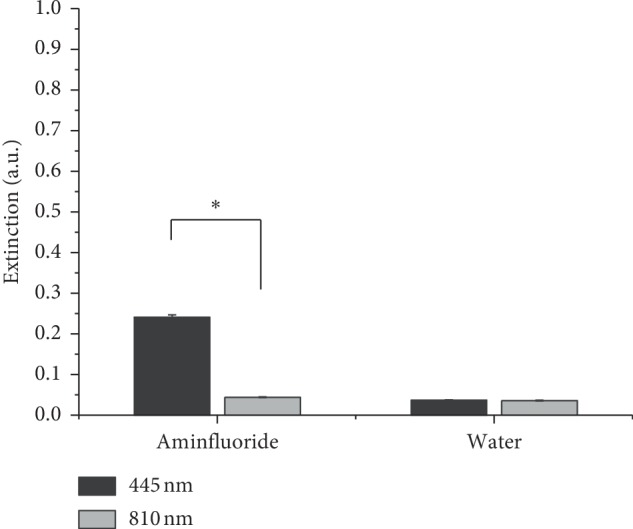
Absorbance of 1% aminfluoride agent (elmex®*fluid*) and distilled water at 445 and 810 nm. The symbol “∗” represents a statistical difference at *p* < 0.05, *n* = 6.

The means of the topical fluoride absorbance at 445 and 810 nm were 0.241 (±0.006) and 0.044 (±0.001) absorbance unit (a.u.), respectively. These results are statistically different (*p* < 0.01) and show that the topical fluoride absorbance at 445 nm is five orders higher than that at 810 nm. The water absorbance of both wavelengths was low and comparable, and its means were 0.036 (±0.001) and 0.037 (±0.001) a.u. at 445 and 810 nm, respectively. Absorbance at a specific wavelength of 1 a.u. indicates that 90% of the incident light is absorbed by the material. Accordingly, the absorbed light by the 1% aminfluoride agent (elmex®*fluid*) at 445 and 810 nm was 21.7% and 4.0%, respectively (Figure 7).

### 3.5. SEM Analysis

**Figure 8:**
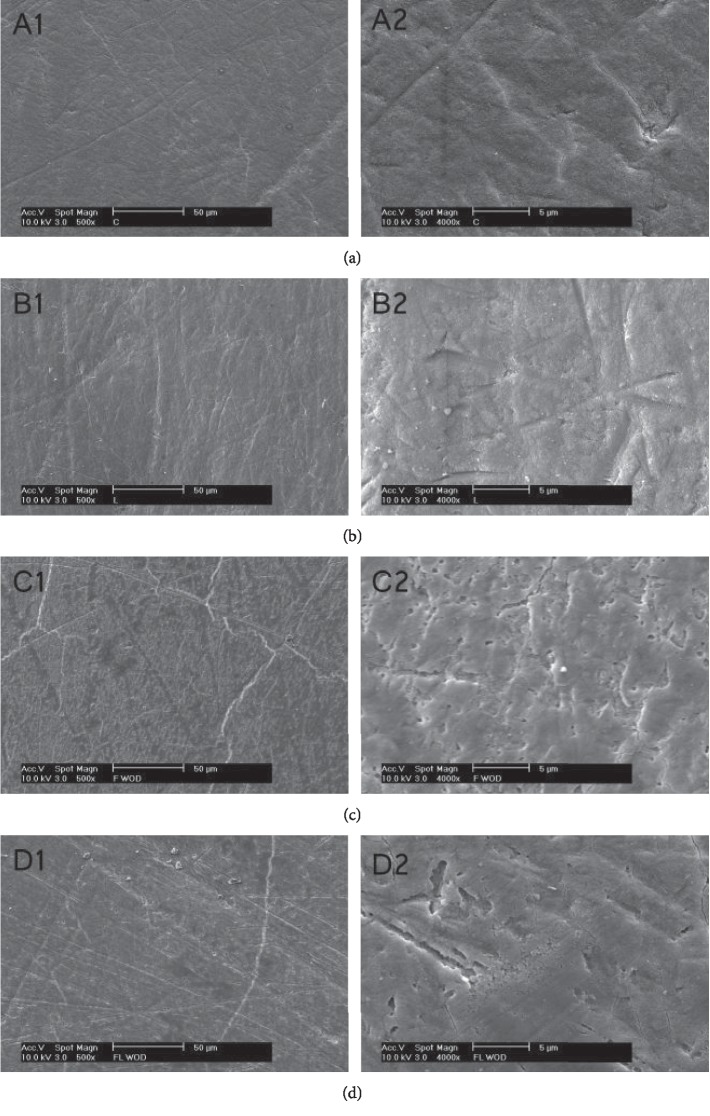
SEM pictures of enamel samples after treatment according to the respective groups. The pictures in the right side are pictures of higher magnification (4000x) in the left side (500x). (a) Control. (b) Laser. (c) Fluoride. (d) Flouride + laser.

SEM pictures of the treatment and control groups are given in Figure 8. Samples from groups F and FL showed a smear layer which could be read as coating or precipitations of fluoride compounds interacting with enamel surface. The smear layer of the FL group seems to be more continuous than that of the F group which contains a homogenous spacing (Figure 8 C1 and D1). The enamel surface in the C group is intact, and no smear layer was seen, and polishing lines were seen scattered on the enamel surface, and some detritus were observed distributed on the enamel surface as a bright and irregular in shape and size. The same observations were seen in the L group, and no negative impact like melting of cracking of the enamel was observed.

## 4. Discussion

Although there is a lack of studies using the 445 nm for reducing enamel demineralization, previous studies in the 1990s and 2000s of the 488 nm emitted from argon ion laser showed an increased enamel resistance to acid challenge. The highest effect was noted during the combination between the topical fluoride and laser irradiation [12, 21].

In this study, the preventive effect of the L group was 9% compared with the matched untreated control group. While for the FL group, it was 27.9% which is 8.5% higher than that of the fluoride-only group.

Several mechanisms explaining the cariostatic effect of laser were mentioned in the literature. Of these mechanisms is the photothermal interaction between the laser and hydroxyapatite, which leads to different effects depending on the temperature reached. At 1200°C, enamel melting will takes place, and at 420°C, a reduced enamel carbonate is expected which leads to reduced enamel permeability, and at 100–200°C, a decreased crystalline water amount can be noted [22]. The other mechanism is the laser interaction with the organic matrix of enamel. As it plays important role in controlling enamel diffusion, the influence of presence of the organic matrix in laser-induced reduction of lesion depth was 55% [23].

Increasing fluoride uptake by enamel during laser irradiation was reported, and the mechanism of increasing fluoride uptake by laser is still not clearly explained. Topical fluorides decrease enamel solubility either by formation of fluoridated hydroxyapatite or CaF_2_. The topical fluoride used in this study has a pH 3.9, and this low pH liberates calcium from enamel which interacts with fluoride to from CaF_2_ on enamel surface. CaF_2_ will release fluoride when subjected to acid attack to form fluoridated hydroxyapatite and enhance enamel remineralization. Fluoride penetration in enamel and formation of fluorhydroxyapatite and CaF_2_ is increased by increasing the fluoride concentration and the time of interaction with the enamel [24]. The time of acidulated fluoride application on tooth surface should be limited to 4 min or less in most fluoride products to avoid damaging the enamel. As with all chemical reactions, temperature increases the kinetic energy, speed, and collision rate of the molecules; thus, it increases the rate of the reaction end product in the given amount of time. The temperature factor will overcome the limitation in the application time of the topical fluoride. In this study, the recorded surface temperature of 39°C (∆*T* = 16.67°C) with irradiation time of 1 min may led to increase in the reaction rate between the topical fluoride and enamel as noted in the PLM results (Figure 2). The SEM pictures in Figure 8 further support this finding that the observed fluoride precipitations on the enamel surface may reduce enamel permeability to acids. The increased fluoride absorption (∼22% of the incident light) at 445 nm will further enhance a higher reaction rate. Few studies reported the temperature-dependent fluoride uptake by the enamel. Baglar et al. [25] found in their study that fluoride uptake by enamel from NaF mouth rinse increased by preheating the mouth rinse from 25°C to 37°C and 43°C, and the fluoride concentration in the mouth rinse was decreased after 1 min to be 2, 1.79, and 1.25 ppm, respectively. Barrancos [26] also reported this finding when the room temperature was increased. Putt et al. [27] while testing the anticariogenic properties of preheated 8% SnF_2_ to 25, 45, 65, and 85°C, they reported that adding energy as heat has led to an increase of fluoride uptake to 2256, 3841, 3721, and 7051 ppm, respectively. In a related field, Yan et al. [28] reported a temperature-related significant increase in the concentrations of fluoride release from glass ionomer cements when samples stored in 4, 37, and 55°C, and these concentrations were 40, 140, and 200 *μ*g/cm^2^, respectively. In this context, in vivo argon ion irradiation on fluoridated enamel resulted in retention of 42.3% of the fluoride after 7 days compared with 12.3% of the fluoridated non-lased enamel [29]. Beside the temperature, a reaction rate needs an activation energy that surpasses a certain level necessary for bonds breaking or forming. Accordingly, the photon energy of the 445 nm laser is 2.8 eV which is higher than the 0.1, 0.4, 1.2, and 1.5 eV of the CO_2_, Er : YAG, Nd : YAG, and 810 nm diode laser, respectively. This fact suggests a possibility of photochemical interaction besides the photothermal interaction. This type of interaction enhances the formation of fluorhydroxyapatite through replacing the carbonate or hydroxyl with fluoride, and this replacement is favorable since it will lead to a more stable molecular orientation [30].

Although both wavelengths have low absorption in hydroxyapatite, 445 nm increased the temperature higher than that of 810 nm by 2.5 folds (Figure 5). This may be due to the higher absorption coefficient of 445 nm by the organic components of enamel. This finding may suggest a photoactivation and/or swelling of the organic component of the enamel responsible for mineral exchange which reduces the demineralization of enamel [31].

The results of this study come into agreement with that of Vlacic et al. [32] who found that the action spectrum of the laser irradiated fluoride includes lasers in the visible region of the electromagnetic spectrum. Furthermore, they are comparable to those of Anderson et al. [10] who reported 29% reduction in lesion depth during the combination of topical fluoride (2.0% NaF) and argon ion laser. Previously, the combined fluoride and argon ion laser treatment has reached 50–55% reduction in lesion depth [12, 21, 33]. However, the methodology of the mentioned studies of Flaitz et al. [12], Hicks et al. [21], Anderson et al. [10], and Wersterman et al. [33] was lacking the fluoride only group, and the comparisons were made with the control and the laser only groups. In the present study, there was 9% decrease in the lesion depth formed after the combined FL treatment compared with the F group, and this decrease was not significant and could not be compared with these studies as explained above. Although it is significant when compared with the control group, results of the L group of the current study achieved lower results (∼9%) compared with those of the previously tested argon ion laser (15–41%), and this could be explained by the difference in wavelengths between the 445 nm and argon ion lasers (488 nm alone or 488 + 514 nm) [10, 33]. It seems that the laser-induced cariostatic effect has its peak in the wavelength of the argon ion laser; in shorter wavelengths (445 nm), this effect may starts to declines. This may be related to the difference in the matching between the irradiation wavelengths and the absorbing chromophores in the enamel between these two lasers. In this context, it is worthy to mention that diode laser offers simple technology, affordable cost, small size, and compact design compared with those of argon ion laser. Lasers have shown the capacity of increasing fluoride preventive action on enamel: TiF_4_ before CO_2_ laser irradiation, Er : YAG laser irradiation associated with NaF application, and APF application followed by Er : YAG irradiation have led to increased acid resistance of enamel [34–36].

As previously reported, there were no available published data for caries prevention using the 445 nm laser; lasing parameters used in the current study were based on preliminary experiments and were close those used previously for the argon ion laser [5, 37]. Because bovine teeth were not subjected previously to manufactured fluoride products like human teeth, they were preferred to be used for this kind of studies for standardization. Additionally, caws have the same nutritional conditions and are slaughtered in a comparable age. To further support this noninvasive laser-supported enamel preventive modality, further investigations using different analytical methods like the cross-sectional scanning electron microscopy and ground sections histology are required.

The current test which tried to simulate the in vivo conditions during the intrapulpal measurement with safe outcomes reported that the maximum temperature was below the injury threshold of the pulp of 5.6°C. On the other hand, due to the absence of the vital pulp, this study cannot give exactly the real temperature of the in vivo situation. Despite that, it was conducted in vitro due to the limitation to have such test in vivo without injuring the pulp.

## 5. Conclusions

Based on the obtained data and under the limitation of this in vitro study, the 445 nm laser irradiation may be useful for caries prevention, and its effectiveness is lower than those previously achieved using the argon ion laser.

## Figures and Tables

**Table 1:** Laser treatment parameters.

Groups	Wavelength (nm)	Defocus (mm)	Power (WCW)^*∗*^	Area (cm^2^)	Pd (W cm^−2^)^*∗∗*^	Time (s)	Deposited energy (J)	Dose (J·cm^−2^)
C	—	—	—	—	—	—	—	—
L	445	15	0.3	0.2	1.5	60	18	90
F	—	—	—	—	—	—	—	—
FL	445	15	0.3	0.2	1.5	60	18	90

^*∗*^Continuous wave. ^*∗∗*^Power density.

**Table 2:** Descriptive statistics of lesion depth (*μ*m) obtained in the polarized light microscopy test.

Groups	*n*	Mean	Std. deviation	Std. error	Minimum	Maximum
C	18	123.48^a^	21.93	5.17	77.25	159.25
L	18	112.33^b^	20.42	4.81	75.00	152.25
F	18	99.58^b,c,e^	30.68	7.23	39.00	160.00
FL	18	89.03^d,e^	30.38	7.27	19.25	135.25

RM-ANOVA with Bonferroni correction test results: means with the different letters are statistically different at *p* < 0.05.

**Table 3:** Descriptive statistics of the surface temperature increase (∆T) in (°C).

Laser	*n*	Mean	Std. deviation	Std. error	Minimum	Maximum
445 nm	10	16.67^*∗*^	4.11	1.30	13.08	26.16
810 nm	10	6.26	0.54	0.17	5.30	7.07

^*∗*^Significantly different when compared with 810 nm diode irradiation by the paired *t* test with *p* < 0.01.

**Table 4:** Descriptive statistics of the intrapulpal temperature increment (∆T) in °C and the corresponding crowns thickness (mm).

	*n*	Mean	Std. deviation	Std. error	Minimum	Maximum
Temperature (∆T°C)	12	2.12^*∗*^	0.66	0.19	0.90	2.97
Thickness (mm)	12	4.10	0.38	0.11	3.50	4.7

^*∗*^One-sample *t* test: the safety of the results is significantly below the 5.6°C threshold at *p* < 0.01.

## Data Availability

The data used to support the findings of this study are included within the article.
